# Upper extremity prosthetic selection influences loading of transhumeral osseointegrated systems

**DOI:** 10.1371/journal.pone.0237179

**Published:** 2020-08-06

**Authors:** Carolyn E. Taylor, Alex J. Drew, Yue Zhang, Yuqing Qiu, Kent N. Bachus, K. Bo Foreman, Heath B. Henninger

**Affiliations:** 1 Department of Orthopaedics, University of Utah, Salt Lake City, Utah, United States of America; 2 Department of Biomedical Engineering, University of Utah, Salt Lake City, Utah, United States of America; 3 DJO Surgical, Austin, Texas, United States of America; 4 Department of Epidemiology, University of Utah, Salt Lake City, Utah, United States of America; 5 Department of Veterans Affairs, University of Utah, Salt Lake City, Utah, United States of America; 6 Department of Physical Therapy and Athletic Training, University of Utah, Salt Lake City, Utah, United States of America; National University of Ireland Galway, Galway, Ireland, IRELAND

## Abstract

Percutaneous osseointegrated (OI) implants are increasingly viable as an alternative to socket suspension of prosthetic limbs. Upper extremity prostheses have also become more complex to better replicate hand and arm function and attempt to recreate pre-amputation functional levels. With more functionality comes heavier devices that put more stress on the bone-implant interface, which could be an issue for implant stability. This study quantified transhumeral loading at defined amputation levels using four simulated prosthetic limb-types: (1) body powered hook, (2) myoelectric hook, (3) myoelectric hand, and (4) advanced prosthetic limb. Computational models were constructed to replicate the weight distribution of each prosthesis type, then applied to motion capture data collected during Advanced Activities of Daily Living (AADLs). For activities that did not include a handheld weight, the body powered prosthesis bending moments were 13–33% (range of means for each activity across amputation levels) of the intact arm moments (reference 100%), torsional moments were 12–15%, and axial pullout forces were 30–40% of the intact case (p≤0.001). The myoelectric hook and hand bending moments were 60–99%, torsional moments were 44–97%, and axial pullout forces were 62–101% of the intact case. The advanced prosthesis bending moments were 177–201%, torsional moments were 164–326%, and axial pullout forces were 133–185% of the intact case (p≤0.001). The addition of a handheld weight for briefcase carry and jug lift activities reduced the overall impact of the prosthetic model itself, where the body powered forces and moments were much closer to those of the intact model, and more complex prostheses further increased forces and moments beyond the intact arm levels. These results reveal a ranked order in loading magnitude according to complexity of the prosthetic device, and highlight the importance of considering the patient’s desired terminal device when planning post-operative percutaneous OI rehabilitation and training.

## Introduction

There are two primary classes of active prosthetic devices used by transhumeral amputees: body powered and externally powered [1]. Body powered devices use a cable system and residual body movements to manually control a terminal device. Externally powered devices rely on battery power and motors to move the prosthesis based on user input from buttons or surface electrodes. These devices have received growing attention from prosthesis developers as they give the user more intuitive and versatile control of the system. The abandonment rate of transhumeral prosthetic devices is up to 60% [2, 3], where the most common reason was “too much fuss” followed by pain, device weight, and short residual limbs not amenable to socket suspension [2]. For veterans with transhumeral amputations, device preference has changed dramatically over time. Within the Vietnam veteran population, the most common prosthesis is a mechanical or body powered device. For Operation Iraqi Freedom and Operation Enduring Freedom veterans, the most common prosthesis is a myoelectric or hybrid device (e.g. body powered elbow with myoelectric terminal device) [2].

In 2009, the Defense Advanced Research Projects Agency (DARPA) launched an initiative to create an upper extremity prosthesis with pre-amputation functional levels and return service member amputees to active duty. From this initiative, DEKA Research and Development Corp and Mobius Bionics released the LUKE arm, featuring up to 10 powered joints. Given the advanced level of functionality, it is one of the heaviest limbs available, and requires the longest training time for patients and therapists [4]. This trend towards complex devices that promise improved functionality is likely to continue as more advanced prosthetic devices enter the market.

Body powered systems terminating in a hook are durable and reliable, but they require non-intuitive compensatory motions to control and a great deal of energy expended by the user. This includes motions like shoulder and chest flexion to actuate a single hand open or closed position. The myoelectric hook has similar overall functionality to a body powered hook with control over only the open or closed position of the terminal device, but they introduce more intuitive and less energy expensive control. The myoelectric hand capitalizes on the control progress of the myoelectric hook but gives the user more dexterity. These devices commonly offer several pre-programmed grip patterns that the user can toggle between with a button. Some even offer different thumb configurations through manual adjustment of the thumb starting position. Beyond this, advanced prostheses like the LUKE Arm offer the highest level of dexterity with limited manual user input. These devices use control mechanisms that do not require the user to manually adjust the hand to perform different grips or thumb positions.

In parallel to prosthesis development, research on percutaneous osseointegrated (OI) endoprostheses for prosthesis attachment has rapidly progressed. By eliminating the socket, these systems bypass problems of poor fit and discomfort and allow for quicker prosthesis don and doff time [5]. In the United States, transfemoral percutaneous OI endoprostheses have undergone FDA evaluation, with one gaining humanitarian use approval (OPRA™, Integrum) and another (POP, DJO Global) undergoing clinical trials (NCT02720159). Systems for use with transhumeral amputations are in development with investigational device exemptions from the FDA, promising increased range of motion and humeral rotation that was previously impaired by sockets.

Osseointegrated implant research has been conducted in animal models looking at the bone [6–10] and soft tissue response [11–13]. Post-operative outcomes have been published for transhumeral OI implanted patients implanted abroad [14–16]. Stenlund *et al*. [16] directly measured transhumeral forces and moments during activity in patients, but this study was limited by large variability in subject comfort levels and device type, which may have resulted in lower applied forces and reduced expectations. Increased activity levels and prosthesis complexity are expected to skew loading scenarios higher in the future.

Drew and Izykowski *et al*. [17] estimated the loads at the bone-implant interface using motion analysis data from non-amputee subjects and inverse dynamics to calculate amputation level-dependent demands since normative range of motion and speed may be regained in high functioning amputees. This study did not examine the impact of different prostheses on these loads. Understanding the loading demands due to the prosthesis is important because gradual return to function is recommended during early rehabilitation to ensure bone ingrowth [14]. Overloading can injure the bone-implant interface or disrupt osseointegration due to excessive endoprosthesis micromotion [18–21]. Without understanding how the choice of prosthesis impacts the bone-implant interface loads, it is difficult to prescribe rehabilitation beyond loading “as tolerated.”

In the present study, the data from Drew and Izykowski *et al*. [17] was modified so subject models reflected the upper extremity mass distribution of an amputee after percutaneous OI endoprosthesis implantation. Models including 4 levels of prosthetic limb complexity, from body powered devices with one degree of freedom to the state-of-the-art advanced prostheses with as many as 6 degrees of freedom, were examined. In the present study complexity was directly related to increased weight, but this may not always be the case where future designs integrate significantly lighter componentry. Upper extremity kinetics for the modeled transhumeral prosthesis systems were calculated at variable amputation levels during *advanced* activities of daily living (AADLs). These data will inform clinicians developing post-operative rehabilitation protocols by highlighting how prosthesis type, coupled with amputation level, changes loads experienced by the transhumeral percutaneous OI endoprosthesis. As a result, activity level restrictions and/or the need for overload protection can be further refined in the context of a patient’s choice of prosthesis type and physical limitations or capabilities.

## Materials and methods

### Source data

Motion capture data from Drew and Izykowski *et al*. [17] were used for the present study. All experimental data is available open access through the following repository: https://zenodo.org/record/1040453 [17]. This included upper extremity motion capture collected on 40 non-amputee subjects performing AADLs. The trunk and right arm were tracked using a 10 camera VICON Motion Analysis system (Vicon Motion Systems Ltd., Oxford, UK). Six of the seven total AADLs from the original study were included: jumping jack, jug lift, underhand ball toss, jogging, rapid internal rotation, and briefcase carry. Elbow fall was excluded because it is dependent on the design of the prosthetic elbow, which varies dramatically between systems. Only locked elbow conditions were included for jogging, jumping jacks, briefcase carry, internal rotation, and underhand toss because not all prostheses have powered elbow joints. Jug lift was also included since subjects were instructed to keep their elbow straight, though it was unconstrained during testing. The intact arm segment was defined from shoulder joint center to elbow joint center, with virtual amputations incrementally along that axis. Virtual amputations were created in the same manner where the mass of the arm segment was divided into four equal segments representing amputations at 25% (proximal), 50%, and 75% (distal) the length of the arm segment corresponding to residual humeral length.

### Prosthetic component specifications

Product information for commercially available upper extremity prosthetic devices from Fillauer® (Chattanooga, TN), Ottobock (Duderstadt, Germany), Mobius Bionics (Manchester, NH), and Össur® (Reykajavik, Iceland) was examined. Length and weight specifications were compiled from an online search of the respective product catalogs. Devices were classified into four categories based on the terminal device as a surrogate for overall complexity: (1) body powered, (2) myoelectric hook, (3) myoelectric hand, and (4) advanced prosthetic limb. There was little variation in length and weight of products within a given category (approximately 3 cm and 50 g, respectively).

One prosthesis from each category was obtained to take more detailed measurements on mass distribution and joint width, from which to create a representative virtual device for each category. The Fillauer® standard canted hook in adult size served as the body powered system. The myoelectric hook used was the U3 Arm from Fillauer® Motion Control (Salt Lake City, UT). The myoelectric hand was selected as the BeBionic from Ottobock. Each system came with a standard elbow built-in to the forearm component, which was included in forearm mass and mass distribution of the system. The advanced prosthetic limb was the LUKE Arm from Mobius Bionics. Though we were unable to obtain the transhumeral system of the LUKE Arm, we were able to take measurements on the transradial system and found missing information from technical specifications available from Mobius Bionics.

### Model modifications

**Fig 1:**
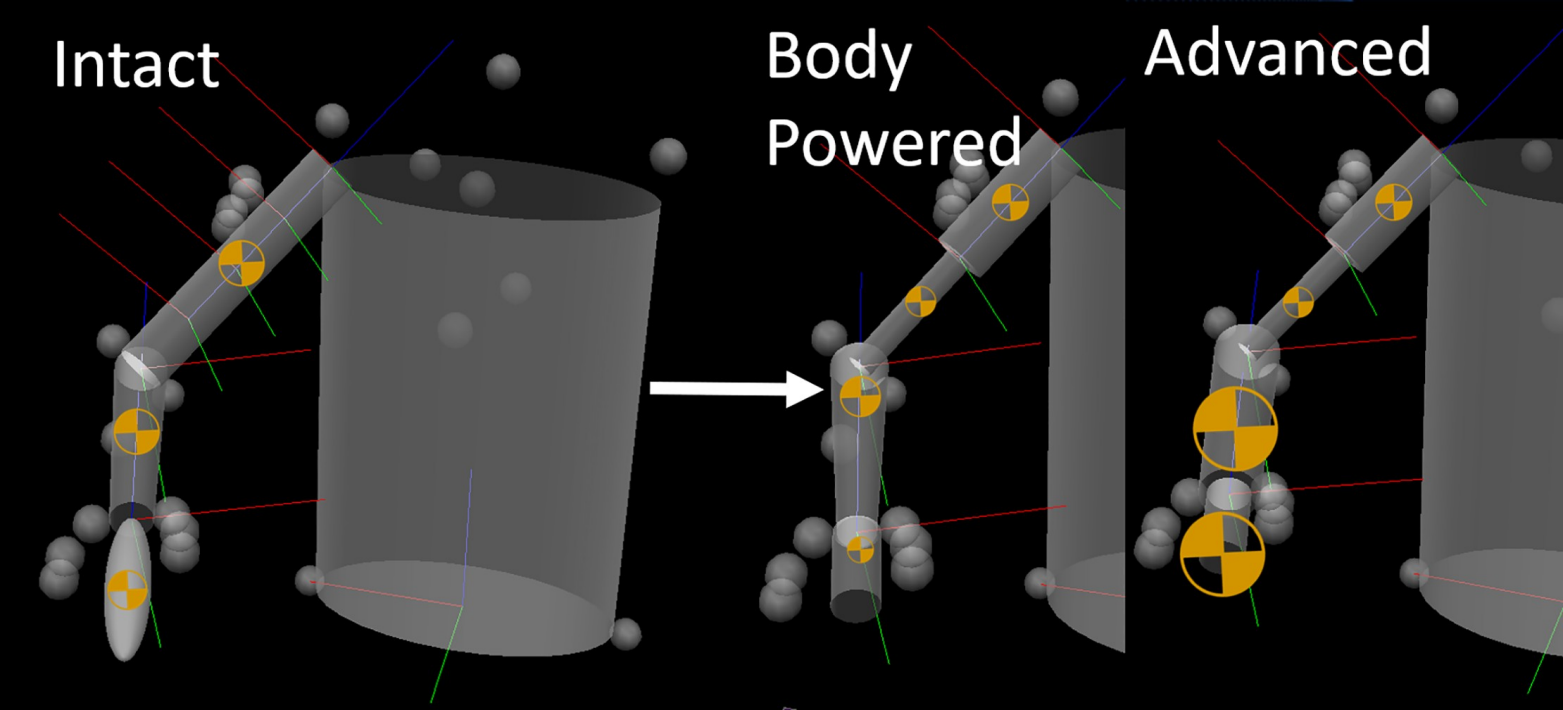
**Model modification from intact (left) to prosthetic models (right).** Model representation in Visual3D of the changes made to the intact for the prosthetic model. A representative 50% amputation level is shown, but 25%, 50%, and 75% amputation levels were created for analysis. ⊕ indicates center of mass and is scaled for segment mass magnitude for the body powered (middle) and advanced (right) prosthetic models.

Due to the relative size agreement between limbs, a single prosthetic arm model from each category was created in Visual3D. This model was then modified to replicate the weight distribution of each category of above elbow prosthesis. Elbow width, forearm mass, forearm center of mass (**COM**), wrist width, hand mass, hand length, and hand COM were altered for each prosthetic arm category (**Table 1**). Each prosthetic arm model was then applied to modify each subject’s anthropometric model (**Fig 1**). Elbow and wrist width were determined from the anthropometric measures of each subject, and then replaced by the respective width of the prosthetic components for the amputation models.

**Table 1:** Metrics of prosthetic arm components for each model.

	Elbow Width (mm)	Forearm Mass (kg)	Wrist Width (mm)	Hand Mass (kg)	Hand Length (mm)
**Body Powered**	63.5	0.555	38.1	0.027	101.6
**Myoelectric Hook**	76.2	1.013	54.0	0.416	152.4
**Myoelectric Hand**	82.5	1.040	63.5	0.500	215.9
**Advanced Prosthesis**	77.3	1.875	63.5	1.525	195.0

Forearm mass included the mass of the integrated elbow components.

Above elbow prostheses typically construct the socket to accommodate for residual limb length and attach directly to the elbow. Percutaneous OI obviates the need for a socket, but no standard elbow interface exists. Residual limb connection proximal to the elbow was modeled as pylons with pyramid attachment, similar to the training prosthesis used in transhumeral percutaneous OI loading studies [16]. Pylon length was customized to the subject’s arm length for 25 and 50% amputation levels. For the most distal 75% amputation level, no pylon was modeled and the prosthetic arm was joined directly to the residual limb with a pyramid adapter model.

### Data processing and statistical analysis

The distribution of kinetic measurements (i.e. bending, torsion and axial) for the different motions were analyzed in the same manner as Drew and Izykowski *et al*. [17] by finding the peak forces and moments then averaging them over three trials. This was completed for each prosthetic model and then compared to the intact model at the different amputation levels (i.e. 25%, 50% and 75%, **Fig 1**). Log transformation of the observed measures created a more approximately normal distribution of the outcomes. A multivariate linear mixed effect regression approach was taken for all comparisons, which accounts for the multiple comparisons issue automatically, to simultaneously model the three force measurements. Comparisons were made to assess the difference between 1) each prosthetic model and the intact model, 2) prosthetic models, and 3) amputation lengths within each model. For each comparison, estimated percent differences in the measurements were calculated between 1) the four prosthetic devices (**Table 1**) and intact model for each combination of motion and amputation level, 2) each pair of prosthetic devices for each combination of motion and amputation level, and 3) each pair of amputation levels for each model and given motion. A total of 810 statistical comparisons were made to capture 4 prosthetic models at 3 amputation lengths for 6 activities.

Finally, a one-sided one-sample Wilcoxon Signed-Rank Test was performed to compare the median of observed loading values to the physical implant loading values from Drew and Taylor *et al* [22]. That study examined yield and ultimate failure of the interface between the bone and a porous coated transhumeral OI endoprosthesis at time-zero before bone ingrowth (not the implant failure itself), both with and without the use of stabilizing screws in axial, torsion, and bending loading modalities for proximal (30%) and distal (65%) amputation levels. Yield values indicate a threshold above which micromotion could initiate at the bone-implant interface and disrupt osseointegration, leading to early post-operative aseptic loosening [19, 20]. Ultimate failure values indicate a threshold where fracture between the bone and endoprosthesis is a dangerous possibility. The modeled 25 and 75% amputation levels were compared to the closest anatomical counterpart: proximal (30% amputation) and distal (65% amputation), respectively. This analysis was performed for all activities and prosthetic models.

Significance level was set at p≤0.05 and all analyses were performed using the statistical programming language R 3.6.3 (2019-07-05) [23]. The main functions applied were ggplot (from ggplot2 package), lme (from nlme package), and other Base R functions.

## Results

Forearm and hand weights were constant in prosthetic models, but intact models varied according to subject-specific anthropometric masses. This increased the relative arm and hand mass in smaller subjects, but decreased them in larger subjects. The smallest subject was a 1.6 m female weighing 44.1 kg, with an anthropometric forearm and hand weighing 0.71 kg and 0.26 kg, respectively. Only the body powered system was lighter than the subject’s native arm (**Table 1**). In contrast, the largest subject was a 1.9 m male weighing 123.7 kg, with an anthropometric forearm and hand weighing 1.98 kg and 0.74 kg, respectively. The forearm of the intact model was always heavier than the prosthesis, but the advanced prosthetic hand was more than double the mass of the intact hand.

For all activities, at all amputation levels, and in all loading modalities, the body powered and advanced prosthesis models were significantly different from the intact model (p≤0.001). All discrete statistical comparisons are provided in **S1 File**, but critical findings are presented in the following text.

Overall maximum bending moment and axial force was observed with the advanced prosthetic model at the 25% amputation level during jumping jacks (58.8±16.8 Nm and 161.9±21.7 N, respectively) (**Table 2**). Overall maximum torsional moment was also observed in the advanced prosthetic model during the internal rotation activity, regardless of amputation level (31.8±14.7 Nm).

**Table 2:** Peak average moments and forces and their associated activity and amputation length for each model.

Mean ± SD		Bending Moment (Nm)			Torsional Moment (Nm)			Axial Force (N)		
**25% Humerus**	**Intact**	Jumping Jack			Internal Rotation			Jumping Jack		
		33.1	±	13.3	19.8	±	11.7	123.8	±	29.4
	**Body Power**	Jumping Jack			Jug Lift			Briefcase Carry		
		22.9	±	2.6	4.3	±	1.8	78.2	±	6.3
	**Myoelectric Hook**	Jug Lift			Internal Rotation			Briefcase Carry		
		25.6	±	2.9	10.0	±	4.7	89.9	±	6.7
	**Myoelectric Hand**	Jumping Jack			Internal Rotation			Briefcase Carry		
		29.8	±	8.5	15.9	±	7.4	94.5	±	7.0
	**Advanced Prosthesis**	***Jumping Jack***			***Internal Rotation***			***Jumping Jack***		
		***58*.*8***	***±***	***16*.*8***	***31*.*8***	***±***	***14*.*7***	***161*.*9***	***±***	***21*.*7***
**50% Humerus**	**Intact**	Jumping Jack			Internal Rotation			Jumping Jack		
		26.3	±	10.3	19.8	±	11.7	106.1	±	26.1
	**Body Power**	Jug Lift			Jug Lift			Briefcase Carry		
		20.1	±	2.3	4.3	±	1.8	77.8	±	6.3
	**Myoelectric Hook**	Jug Lift			Internal Rotation			Briefcase Carry		
		22.3	±	2.5	10.0	±	4.7	89.5	±	6.7
	**Myoelectric Hand**	Jumping Jack			Internal Rotation			Briefcase Carry		
		24.4	±	6.8	15.9	±	7.4	94.1	±	7.0
	**Advanced Prosthesis**	Jumping Jack			***Internal Rotation***			Jumping Jack		
		48.4	±	13.7	***31*.*8***	***±***	***14*.*7***	160.9	±	21.8
**75% Humerus**	**Intact**	Jumping Jack			Internal Rotation			Briefcase Carry		
		20.7	±	7.8	19.8	±	11.7	92.8	±	10.5
	**Body Power**	Jug Lift			Jug Lift			Briefcase Carry		
		17.4	±	2.0	4.3	±	1.8	75.8	±	6.1
	**Myoelectric Hook**	Jug Lift			Internal Rotation			Briefcase Carry		
		19.1	±	2.2	10.0	±	4.7	87.5	±	6.6
	**Myoelectric Hand**	Jug Lift			Internal Rotation			Briefcase Carry		
		20.1	±	2.3	15.9	±	7.4	92.1	±	6.9
	**Advanced Prosthesis**	Jumping Jack			***Internal Rotation***			Jumping Jack		
		38.9	±	10.7	***31*.*8***	***±***	***14*.*7***	155.4	±	21.2

Global maxima are highlighted in ***bold italics***.

### Intact versus prosthetic models

Activities with a handheld weight (briefcase carry and jug lift) and without (jogging, jumping jack, internal rotation, and underhand toss) were impacted differently by the change in weight added distally. During unweighted activities, the body powered prosthesis had decreased bending moments, torsional moments, and axial pullout forces as compared to the intact model (p≤0.001) (**Table 3**). The myoelectric hook showed differences from the intact model in all comparisons except torsion during jogging (p = 0.140). The myoelectric hand model showed the most overall similarity to the intact model with a slight decrease in bending and torsional moments and axial pullout force, except during jogging where torsional moment increased and axial pullout force increased at the 75% amputation level. The advanced prosthesis increased bending moments, torsional moments, and axial pullout forces versus the intact model (p≤0.001). For weighted activities, the same trends held for the body powered and advanced prosthesis but the percent change decreased versus the unweighted activities (p≤0.001). Here, the myoelectric hand model maintained the most similarity to the intact model.

**Table 3:** Difference between intact and prosthetic models.

	**Bending**	**Torsion**	**Axial**
	**Unweighted**		
**Body Powered**	13.2–33.3% *	12.0–15.3% *	29.9–39.7% *
**Myoelectric Hook**	59.8–73.1% *	51.6–93.7%	61.9–82.9% *
**Myoelectric Hand**	89.1–98.8%	44.3–97.4%	75.0–100.7%
**Advanced Prosthesis**	177.2–201.4 % *	163.5–325.5% *	133.4–185.1% *
** **	**Weighted**		
**Body Powered**	82.9–91.9% *	90.1–90.5% *	73.7–82.0% *
**Myoelectric Hook**	92.6–97.4% *	97.2–97.5% *	85.2–94.6% *
**Myoelectric Hand**	97.6–101.7%	101.8–102.3% *	89.9–99.9%
**Advanced Prosthesis**	112.9–118.7% *	116.7–119.8% *	110.8–125.6% *

Reported values are the range of percent difference of prosthetic models compared to the intact model (100%) across all activities and amputation levels.

* indicates statistical signifiance between the intact and prosthetic model (p≤0.030)

### Differences between prosthetic models

**Fig 2:**
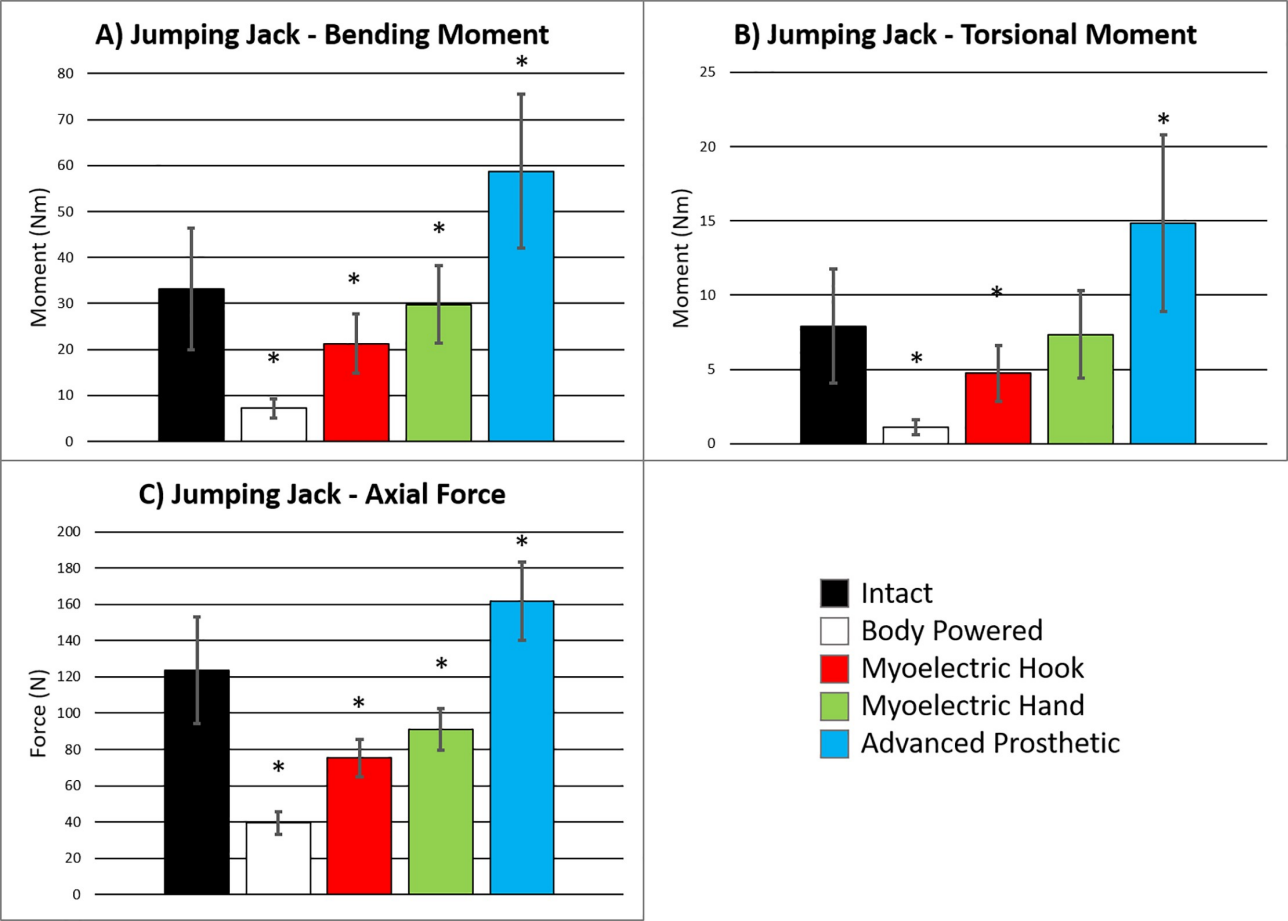
Peak moments and forces for 25% amputation levels in bending, torsional, and axial directions. **A)** Bending moment for body powered and myoelectric hook models were lower than intact, whereas the advanced prosthetic arm bending moment was higher. **B)** The same pattern held true in torsional moment across models. **C)** Axial force was lower for body powered, myoelectric hook, and myoelectric hand models compared to the intact and higher for the advanced prosthetic arm model compared to the intact model. * *indicates significant difference compared to the intact model (p≤0*.*025)*.

Comparisons between prosthetic models revealed differences between all models, where the largest difference was observed between the body powered and advanced prostheses and the smallest difference between the myoelectric hook and hand (p≤0.001). There was a clear ranked order of loads associated with prosthesis complexity (**Fig 2**). Each prosthetic model showed the same trend in amputation length as the intact model, where 25% amputation levels showed the highest peak moments and forces [17]; however, the activity that showed the highest peak moment and force was not always consistent (**Table 2**).

### Effect of amputation level

When comparing amputation levels, differences were seen between all levels in bending for all activities except jogging and briefcase carry. During jogging, the advanced prosthesis did not show differences between the 25 and 75% levels (p = 0.953) and the myoelectric hook did not show differences between the 50 and 75% levels (p = 0.934). During briefcase carry, models were different only between the 25 and 50% amputation levels (p≤0.006) and only the advanced prosthesis was different between the 25 and 75% levels (p<0.001). Torsional moments did not show amputation level dependence for any activities or models. Axial pullout forces were more variable in their dependence on amputation level. There was no difference between the 25 and 50% levels for all activities and prosthetic models except the myoelectric hook during jumping jack and jug lift (p≤0.045) and the body powered during jumping jack, jug lift, and internal rotation (p≤0.023). For the 25 vs 75% comparisons, there was difference for all activities and prosthetic models except the advanced prosthesis during briefcase carry (p = 0.112). For the 50 vs 75% comparisons, there were differences for all activities except internal rotation for the advanced prosthesis (p = 0.056) and briefcase carry for myoelectric hook, hand, and advanced prosthesis (p≥0.050).

### AADL loading versus implant failure

In comparisons between the present data and the mean yield and ultimate failure of a transhumeral OI implant [22], there were no cases where modeled axial pullout exceeded yield (784.2–1818.1 N) or ultimate failure (1325.1–5120.3 N). In bending, all activities and prosthetic models exceeded mechanical yield (1.7–2.3 Nm) except the body powered model during internal rotation, jogging, and underhand toss at the distal amputation level. Comparison to bending ultimate failure did not result in any loading that exceeded mechanical tests (70.3–119.4 Nm). Under torsional loading, the advanced prosthesis during internal roation and jumping jack, and myoelectric hand during internal rotation, exceeded yield at the proximal and distal level (4.5–10.2 Nm). Additionally, the myoelectric hand during jumping jack, and hook during internal rotation, exceeded yield at the proximal level when compared to implants without screws for stabilization (4.5 Nm). Torsional loading also exceeded ultimate failure for the advanced prosthesis during jumping jack and internal rotation, and for the myoelectric hand during internal rotation at the proximal and distal level only without screws (6.0–12.7 Nm). The myoelectric hand during jumping jack and myoelectric hook during internal rotation also exceeded proximal ultimate failure for implants without screws for stabilization (6.0 Nm). The only condition that was not at risk for any prosthetic models, activities, and loading modalities was ultimate failure at the proximal and distal levels when compared to implants with the use of stabilzing screws. It is important to note that all these loading values reported for the bone-implant interface are far below the minimum expected implant failure threshold for the implant tested in Drew et al. [22] (~28 kN shear load to fracture for the smallest implant size, data not shown, esimtated from FE models of the implant design).

## Discussion

The purpose of this study was to examine the impact of prosthesis type on the estimated forces at the bone-implant interface of a transhumeral percutaneous OI endoprosthesis. We analyzed the contributions of prosthesis type and residual limb length, and compared each case to failure testing data from a transhumeral percutaneous OI system [22] to determine which loading scenarios exceeded time-zero implant stability, above tested yield and ultimate failure limits. Only the advanced prosthetic limb increased overall loading compared to the intact limb, but all prosthetic models changed the estimated loading during AADLs. Amputation level was a major factor in bending and axial loading directions, with the largest impact on bending. Finally, these data supported the use of bicortical screws to improve torsional stability when compared to ultimate failure of a transhumeral percutaneous OI endoprosthesis [22], since this was only scenario where all analyzed activities could be completed with any prosthesis at any amputation level compared to laboratory test data. These findings stress the importance of 1) utilizing accurate prosthesis weight distribution in motion capture and load estimates for amputees, 2) considering desired prosthesis and residual limb length when prescribing rehabilitation, and 3) the risk of mechanical compromise when completing AADLs with prostheses during the early post-operative period.

Jumping jacks at the 25% amputation level had the highest peak forces and moments in the intact model. The advanced prosthetic arm increased bending moments 1.8x, torsional moments 1.6x, and axial forces 1.3x compared to the intact condition (**Fig 2**). All other prosthetic models were either comparable or decreased the peak forces and moments as compared to the intact model. Comparisons between the prosthetic models revealed the importance of modeling the correct prosthesis type. There was a clear ranked order of forces and moments, paralleling the order of complexity of the device. While it was not surprising that the heaviest device resulted in the highest loads, these results indicate there should be a gradual increase in prosthesis weight by percutaneous OI implant patients. For example, a pre-operative advanced prosthesis user should spend more time in a training prosthesis that uses lighter and simpler components that allows them to better control load on the bone-implant interface before transitioning to heavier and more functional take-home systems compared to a patient who uses a body powered system. Similarly, there should be a training period before moving to a more complex system post-operatively. If a patient with a myoelectric hand desires an advanced prosthesis, they should train with added weight on the existing device before permanently transitioning. This would ensure the patient’s tolerance of the loading is assessed conservatively, building up toward higher levels of personal and device functionality.

The impact of amputation level on loading magnitude was also pronounced. In bending, a 25% reduction in residual limb length resulted in up to a 46.5% increase in moments. In axial pullout, a 25% reduction in amputation level resulted in up to a 17.8% increase in forces. Increased loading with shorter residual limbs can be attributed to the longer moment arm and mass distal to the amputation site as was similarly observed in Drew et al. [17]. Historically, all amputation levels have been treated the same with respect to percutaneous OI rehabilitation [15, 24]. Our results indicate that shorter residual limbs should be approached with caution, since bone-implant interface failure becomes catastrophic as the chance for revision surgery decreases with shorter residual bone. Prosthetists should consider more time in a training prosthesis before independent prosthesis use for patients with shorter residual limbs.

The present data provide an estimate of the maximum daily loads a transhumeral amputee with percutaneous OI attachment will impart on the bone-implant interface after full recovery and stable bone ingrowth. During recovery, patients are restricted in their movement and loading until approximately 6 months after surgery [25], meaning the dynamic movements analyzed herein would not be permitted during early recovery. Though it is unlikely that these maximum daily loads will ever be imparted on the bone-implant interface with no stabilizing bone ingrowth, comparing these data to cadaveric time-zero mechanical testing represents an absolute worst-case condition. This comparison reveals an alarming overlap between daily loading and loads to failure of the bone-implant interface during the early post-operative period (**Table 4**) [22, 26]. The range of axial pullout yield load (784.2–1818.1 N) [22] was at least 4.8x the maximum estimated axial force (161.9 ± 21.7 N). This suggests that axial micromotion is unlikely during the early post-operative period due to AADL loading, but accidental overloading events could exceed these thresholds.

**Table 4:** Activity loads compared to failure loads reported in the literature.

Study	Reported	Bending (Nm)	Torsional (Nm)	Axial Force (N)
**Active Loading**				
**Drew & Izykowski *et al*. [17]**	Maximum activity loads	40.7 ± 9.4	24.9 ± 11.2	138.7 ± 21.4
**Stenlund *et al*. [16]**	Maximum reported value	37.2 ± 2.9	15.6 ±3.4	109.1 ± 7.2
**Advanced Prosthetic Model**	Maximum activity loads	58.8 ± 16.8	31.8 ± 14.7	161.9 ± 21.7
**Failure Testing**				
**Drew & Taylor *et al*. [22]**	Yield Mean Range	*1*.*7–2*.3*	*4*.*5–10*.2*	784.2–1,818.1
**Drew & Taylor *et al*. [22]**	Ultimate failure mean range	70.3–119.4	*6*.*0–42*.2*	1,325.1–5,120.3
**Welke *et al*. [26]**	Ultimate failure load	*36*.*7 ± 11*.0*	NA	NA

Both failure studies used a cementless press-fit intramedullary stem. Drew & Taylor et al. [22] tested a transhumeral porous coated implant for percutaneous OI attachment (DJO Surgical, Austin, Texas). Welke et al. [26] tested an established cementless intramedullary stem (MUTARS Implantcast, Germany).

* Overlap between failure and daily loading values

Torsional and bending loading regimes pose a greater risk. In bending, yield load (1.7–2.3 Nm) [22] could result in unsafe loading for both amputation levels. Ultimate failure loads (70.3–119.4 Nm) [22] were at least 1.2x the maximum estimated loads (58.8 ± 16.8 Nm) (**Table 4**). This indicates that micromotion from bending loads is a concern early post-operatively, and the small margin of error between daily loading and ultimate failure should not be ignored. Micromotion is a more pronounced concern in torsional loading where there is a glaring overlap between both yield loads (4.5–10.2 Nm) and ultimate failure (6.0–42.2 Nm) [22] and maximum estimated loading (31.8 ± 14.7 Nm). Though yield loading is not an acute concern like ultimate failure (fracture), sustained micromotion can lead to surgical implant revision and should be approached with just as much caution as fracture. Though for the implant design examined herein, failure of the implant itself would not occur anywhere near these thresholds, it is important to consider for future percutaneous OI implant designs.

These results from cadaveric studies represent a worst case early post-operative period where there is little or no stabilizing bone ingrowth into the percutaneous OI endoprosthesis. Progressive bone ingrowth should increase failure thresholds as evidenced by a sheep implant retrieval study [9]. This study saw a 14x increase in mechanical pullout force after 12 months compared to time zero. Currently there is no established method to asses ingrowth progression in humans, but animal models indicate steady progression over the first 12–24 months following implantation if initial stability is satisfied and micromotion is mitigated [7, 9, 10].

It is difficult to dictate what forces are unsafe for AADL loading in a percutaneous OI patient without data characterizing the rate of bone ingrowth in humans or failure thresholds after bone ingrowth has occurred. However, voluntary loading data collected on transhumeral amputees with OI attachment shows that loading above these levels is possible without failure, at least one year post-implantation (**Table 4**) [16]. With data from animal studies and evidence of successful loading in transhumeral OI systems, we expect the risk of failure to decrease over time, but do not know how fast or by how much. Beyond the early post-operative time period, activities with a high bending and torsional moment should be approached with caution until empirical evidence to the contrary is collected in living patients. One proposed mitigation solution is to implement external breakaway devices to protect patients from bone-implant interface failure. These systems should fail at predictable loads below initial stability yield values, especially in bending and torsion, but could be increased as patient tolerance and clinical evidence supports safe use.

Rehabilitation for percutaneous OI users has relied on patient tolerance and the use of training prostheses early postoperatively [14] with little guidance on patient specific techniques that accommodate prosthesis type and residual limb length. The present results suggest that this rehabilitation should be further refined by prosthesis type and residual limb length. A patient intending to use a heavier prosthesis or with shorter residual limb may require a longer more rigorous rehabilitation protocol before being allowed to complete the same baseline activities as someone who uses a lighter prosthesis or has a longer residual limb due to their inherent loading of the percutaneous OI endoprosthesis.

The data presented herein could also assist device designers who wish to protect amputees with percutaneous OI attachment. Changing the location of the mass and COM of the forearm and hand segments plays a role in peak loading of the simulated bone-implant interface. In order to determine how these two metrics independently impacted loading, we selected one average subject’s motion and altered the mass of the forearm and hand each by 0.5 and 1.5x. We found this altered peak loading magnitudes by 0.5 and 1.5x for all scenarios, with the exception of the body powered system where the relationship was 0.7 and 1.3x, respectively. We then altered the COM of the forearm and hand segments so they were located 50% proximal and 50% distal to the actual COM location. This resulted in a smaller shift proximally because the COM was originally located proximal of center for all prostheses. The shift in COM had the smallest influence on axial forces with a 1.0–1.1x increase with more distal COM. A proximal shift of the COM had little effect on torsional and bending moments (versus a 1.0–1.3x increase with distal movement), but the greatest effect was seen for the body powered system (1.3 and 1.2x increase in torsion and bending, respectively). Shifting the COM distally resulted in the greatest change especially in torsion where moments increased 1.3–2.4x, with the body powered system experiencing the greatest change. In bending, the increase was 1.4–1.7x with distal shift of the COM. This single subject analysis indicates that moving the COM of a body segment has a bigger effect on peak loading than reducing the weight, which should be considered in future prosthetic system designs to reduce bone-implant loading.

The primary limitation of this study is that the input motion data was collected on non-amputee volunteers. These motion patterns may change dramatically when completed by an amputee with an upper extremity prosthesis connected by either a socket or percutaneous OI attachment, which would then alter the estimated loading of the bone-implant interface. Such compensation strategies are difficult to predict as they are based on level of amputation, comorbidities, prosthesis selection, proficiency of use, and other factors not captured here [27]. The goal of percutaneous OI attachment is to return patients to full range of motion and allow them to complete these activities with reduced compensation. Therefore, these motions represent a best-case scenario for high functioning amputees where percutaneous OI implant systems allowed them to regain full activity, and estimated the worst-case scenario of generating maximum possible loads transferred to the bone. This also assumes that the individual still has full shoulder range of motion and the ability to use all types of prostheses. Future research is necessary to determine exactly how a transhumeral amputee with percutaneous OI attachment would alter limb kinematics during these motions. While it was beyond the scope of this study due to the lack of comprehensive motion capture data for the transhumeral amputation population, and specifically individuals using various prosthetic systems, it is reasonable to assume that heavier systems could slow the speed and alter the range of motion, affecting resultant bone-implant interface forces. Additionally, only a small subset of upper extremity prostheses were modeled for this study. This study did not capture all the possible devices an amputee may utilize and did not take into account the adaptive devices that amputees use to facilitate ADLs and AADLs. However, this study has modeled the major categories of devices that could affect skeletal loading. As OI attachment systems become more widely available and users adapt to having the systems, the demands placed on the bone-implant interface will also grow. Our study modeled a limited number of activities and does not capture every loading scenario that may come in the future, but provides an assessment of loading expected in the first wave of AADL users of percutaneous OI implants.

In conclusion, transhumeral percutaneous OI implants have the potential to maximize upper extremity range of motion and eliminate the constraints of a socket. Higher demand activities would then impart more force on the bone-implant interface. Individuals may also choose to upgrade their current prosthetic systems to include more complex functionality. These benefits may result in a greater risk for periprosthetic fracture, especially during the early post-operative period when bone ingrowth is absent or minimal. The present study has: 1) demonstrated the need to model specific prosthetic devices when estimating loading conditions at the bone-implant interface of a percutaneous OI endoprosthesis, 2) showed that shortening residual limb length and increasing complexity of prosthetic devices increases overall load on the bone-implant interface, and 3) determined that AADL loading, especially in bending and torsion, can put percutaneous OI patients at risk for excess micromotion and fracture in the early post-operative period. These results will better inform rehabilitation protocols based on patient specific factors and goals to help protect future upper extremity amputees with percutaneous OI attachment.

## Supporting information

S1 File (DOCX)
