# Supplementary material for: Upper extremity prosthetic selection influences loading of transhumeral osseointegrated systems
Source: PLoS One. 2020 Aug 6;15(8):e0237179. doi: 10.1371/journal.pone.0237179 (PMC7410272; doi:10.1371/journal.pone.0237179)
Supplement: S1 File — (DOCX) [file pone.0237179.s001.docx]

# Intact versus prosthetic model use

Hypothesis: Prosthetic model kinetics will be significantly different from the intact model indicating motion capture data models for amputees should be modified to reflect prosthetic components.

**Table 1. Percentage Change in Force Measurement (Models Compared to Intact Arm)**

|  |  | 25% | | | 50% | | | 75% | | |
| --- | --- | --- | --- | --- | --- | --- | --- | --- | --- | --- |
| Motion | Model | Bending% (CI%) | Torsion% (CI%) | Axial% (CI%) | Bending% (CI%) | Torsion% (CI%) | Axial% (CI%) | Bending% (CI%) | Torsion% (CI%) | Axial% (CI%) |
| Briefcase Carry | Body Powered | -10 (-11.7, -8.2)*** | -9.5 (-11.3, -7.7)*** | -25.7 (-27.1, -24.2)*** | -8.1 (-9.6, -6.5)*** | -9.5 (-11, -7.9)*** | -21.3 (-22.7, -20)*** | -9.2 (-10.6, -7.9)*** | -9.5 (-10.8, -8.1)*** | -18 (-19.3, -16.8)*** |
|  | Myoelectric Hook | -3.7 (-5.5, -1.7)*** | -2.6 (-4.5, -0.6)** | -14.5 (-16.1, -12.8)*** | -2.6 (-4.2, -0.9)** | -2.6 (-4.2, -0.9)** | -9.4 (-11, -7.9)*** | -3.7 (-5.1, -2.2)*** | -2.5 (-4, -1)*** | -5.4 (-6.8, -3.9)*** |
|  | Myoelectric Hand | 0.7 (-1.3, 2.7) | 2.2 (0.2, 4.3)* | -10.1 (-11.9, -8.3)*** | 1.7 (-0.1, 3.4) | 2.3 (0.5, 4)* | -4.8 (-6.4, -3.1)*** | 0.6 (-0.9, 2.1) | 2.3 (0.8, 3.8)** | -0.4 (-1.9, 1.1) |
|  | Advanced Prosthetic | 17.6 (15.3, 19.9)*** | 19.7 (17.4, 22.1)*** | 10.8 (8.7, 13)*** | 15.1 (13.1, 17.1)*** | 19.8 (17.7, 21.8)*** | 17.5 (15.5, 19.5)*** | 12.9 (11.2, 14.6)*** | 19.8 (18, 21.6)*** | 23.4 (21.5, 25.2)*** |
| Jumping Jack | Body Powered | -77.3 (-78.5, -76.1)*** | -85.4 (-86.2, -84.6)*** | -67.6 (-69.3, -65.8)*** | -78.7 (-79.8, -77.5)*** | -85.4 (-86.2, -84.6)*** | -63.2 (-65.1, -61.1)*** | -82.4 (-83.4, -81.4)*** | -85.4 (-86.2, -84.6)*** | -62.1 (-64.2, -60)*** |
|  | Myoelectric Hook | -33 (-36.5, -29.3)*** | -37.2 (-40.5, -33.7)*** | -38 (-41.3, -34.6)*** | -32.6 (-36.2, -28.8)*** | -37.2 (-40.5, -33.6)*** | -28.6 (-32.4, -24.5)*** | -34.5 (-38, -30.8)*** | -37.1 (-40.5, -33.5)*** | -19.8 (-24.2, -15.3)*** |
|  | Myoelectric Hand | -6 (-11, -0.8)* | -2.8 (-7.9, 2.6) | -25 (-29, -20.9)*** | -3.4 (-8.6, 2) | -2.7 (-7.9, 2.8) | -13.4 (-18, -8.5)*** | -2.5 (-7.8, 3) | -2.6 (-7.9, 2.9) | -1.7 (-7, 3.9) |
|  | Advanced Prosthetic | 85.4 (75.6, 95.7)*** | 97 (86.6, 107.9)*** | 33.4 (26.4, 40.8)*** | 91.7 (81.5, 102.5)*** | 97.2 (86.7, 108.3)*** | 54.8 (46.6, 63.5)*** | 96 (85.4, 107.1)*** | 97.4 (86.7, 108.6)*** | 81 (71.2, 91.3)*** |
| Jug Lift | Body Powered | -17.1 (-18.3, -15.8)*** | -9.9 (-11.2, -8.5)*** | -26.3 (-27.3, -25.2)*** | -14.9 (-16.1, -13.7)*** | -9.9 (-11.1, -8.6)*** | -21.9 (-23, -20.8)*** | -13.3 (-14.4, -12.1)*** | -9.9 (-11.1, -8.7)*** | -18.6 (-19.6, -17.5)*** |
|  | Myoelectric Hook | -7.4 (-8.8, -6)*** | -2.8 (-4.2, -1.4)*** | -14.8 (-16, -13.5)*** | -5.7 (-7, -4.4)*** | -2.8 (-4.2, -1.4)*** | -9.6 (-10.9, -8.4)*** | -4.9 (-6.1, -3.6)*** | -2.8 (-4.1, -1.5)*** | -5.5 (-6.7, -4.2)*** |
|  | Myoelectric Hand | -2.4 (-3.8, -1)** | 1.8 (0.3, 3.3)* | -10.1 (-11.4, -8.7)*** | -0.7 (-2.1, 0.7) | 1.8 (0.4, 3.3)* | -4.6 (-6, -3.3)*** | 0.1 (-1.2, 1.5) | 1.8 (0.5, 3.2)** | -0.1 (-1.4, 1.2) |
|  | Advanced Prosthetic | 17.7 (16, 19.5)*** | 16.7 (15, 18.4)*** | 12.5 (10.8, 14.2)*** | 18.7 (17, 20.3)*** | 16.7 (15.1, 18.3)*** | 19.4 (17.7, 21.1)*** | 18 (16.5, 19.6)*** | 16.7 (15.1, 18.2)*** | 25.6 (23.9, 27.2)*** |
| Jogging | Body Powered | -66.7 (-69.4, -63.7)*** | -85 (-86.2, -83.6)*** | -66.4 (-69.2, -63.4)*** | -74.4 (-76.5, -72.1)*** | -85 (-86.2, -83.6)*** | -61.7 (-64.9, -58.2)*** | -86.8 (-87.9, -85.6)*** | -85 (-86.3, -83.7)*** | -60.3 (-63.6, -56.8)*** |
|  | Myoelectric Hook | -26.9 (-33, -20.4)*** | -6.3 (-14, 2.1) | -36.1 (-41.4, -30.3)*** | -33.1 (-38.7, -27)*** | -6.3 (-14.1, 2.2) | -26.2 (-32.3, -19.5)*** | -40.2 (-45.1, -34.8)*** | -6.3 (-14, 2.1) | -17.1 (-23.9, -9.7)*** |
|  | Myoelectric Hand | -2.7 (-10.7, 6) | 55.6 (42.8, 69.6)*** | -23.6 (-29.9, -16.7)*** | -1.2 (-9.5, 7.7) | 55.6 (42.7, 69.8)*** | -11.6 (-18.9, -3.6)** | -5.7 (-13.5, 2.7) | 55.7 (42.9, 69.6)*** | 0.7 (-7.6, 9.7) |
|  | Advanced Prosthetic | 91 (75.2, 108.1)*** | 225.5 (198.6, 254.7)*** | 35.7 (24.5, 47.9)*** | 101.4 (84.6, 119.6)*** | 225.5 (198.5, 255)*** | 57.8 (44.7, 72.1)*** | 95.1 (79.1, 112.6)*** | 225.5 (198.7, 254.7)*** | 85.1 (69.8, 101.6)*** |
| Underhand Toss | Body Powered | -78.6 (-79.8, -77.3)*** | -84.7 (-85.6, -83.8)*** | -70.1 (-71.9, -68.3)*** | -80.7 (-81.8, -79.5)*** | -84.7 (-85.5, -83.7)*** | -67.2 (-69.1, -65.2)*** | -84 (-84.9, -83)*** | -84.7 (-85.6, -83.8)*** | -67.3 (-69.2, -65.4)*** |
|  | Myoelectric Hook | -36.6 (-40.2, -32.7)*** | -39.2 (-42.7, -35.4)*** | -37 (-40.6, -33.1)*** | -37.2 (-40.8, -33.4)*** | -39 (-42.5, -35.3)*** | -30.7 (-34.6, -26.4)*** | -39.4 (-42.9, -35.8)*** | -38.9 (-42.4, -35.2)*** | -25.5 (-29.8, -21)*** |
|  | Myoelectric Hand | -10.4 (-15.6, -4.9)*** | -5.6 (-11, 0.2) | -21 (-25.6, -16.2)*** | -9.2 (-14.5, -3.7)** | -5.4 (-10.8, 0.4) | -13.1 (-18.1, -7.8)*** | -9.4 (-14.6, -3.9)** | -5.1 (-10.5, 0.6) | -5.5 (-10.9, 0.3) |
|  | Advanced Prosthetic | 77.2 (67, 88)*** | 91.1 (80.1, 102.8)*** | 46.3 (37.8, 55.2)*** | 80.4 (70.1, 91.4)*** | 91.5 (80.5, 103.2)*** | 61 (51.7, 70.8)*** | 82.1 (71.7, 93.1)*** | 91.9 (81, 103.5)*** | 78.7 (68.5, 89.5)*** |
| Internal Rotation | Body Powered | -80.5 (-81.5, -79.5)*** | -87.4 (-88, -86.7)*** | -69.7 (-71.2, -68)*** | -80.1 (-81.2, -78.8)*** | -88 (-88.7, -87.2)*** | -66.6 (-68.5, -64.5)*** | -81.4 (-82.4, -80.4)*** | -87.3 (-88, -86.6)*** | -67.2 (-69, -65.4)*** |
|  | Myoelectric Hook | -38.3 (-41.5, -35)*** | -45.8 (-48.6, -42.9)*** | -38.1 (-41.3, -34.8)*** | -37.2 (-40.9, -33.3)*** | -48.4 (-51.4, -45.2)*** | -30.6 (-34.7, -26.3)*** | -37.3 (-40.6, -33.7)*** | -45.7 (-48.6, -42.7)*** | -25.1 (-29.1, -20.9)*** |
|  | Myoelectric Hand | -10.9 (-15.4, -6.1)*** | -14.2 (-18.6, -9.6)*** | -22 (-26, -17.8)*** | -9.1 (-14.4, -3.4)** | -18.3 (-23.1, -13.2)*** | -12.3 (-17.4, -6.9)*** | -6.1 (-11.2, -0.8)* | -14.1 (-18.7, -9.2)*** | -3.7 (-8.9, 1.7) |
|  | Advanced Prosthetic | 79.1 (69.9, 88.7)*** | 71.7 (62.9, 80.9)*** | 44.1 (36.7, 51.8)*** | 82.2 (71.5, 93.5)*** | 63.5 (53.9, 73.7)*** | 62.8 (53.3, 72.9)*** | 89 (78.8, 99.7)*** | 70.5 (61.4, 80.1)*** | 82.2 (72.4, 92.5)*** |

Notes: The percentage change in force measurement between current model and intact arm is reported;
95% Confidence Intervals of percentage changes are reported in parentheses;
$***$ indicates that p-value < 0.001; $**$ indicates that $0.001\leq p-value<0.01$; $*$ indicates that $0.01\leq p-value<0.05$.

# Difference between prosthetic models

Hypothesis: There is a ranked order in complexity that correlates to increased forces imparted on the bone-implant interface.

**Table 2. Percentage Change in Force Measurement (Models Pairwise Comparison)**

|  |  | 25% | | | 50% | | | 75% | | |
| --- | --- | --- | --- | --- | --- | --- | --- | --- | --- | --- |
| Motion | Model | Bending% (CI%) | Torsion% (CI%) | Axial% (CI%) | Bending% (CI%) | Torsion% (CI%) | Axial% (CI%) | Bending% (CI%) | Torsion% (CI%) | Axial% (CI%) |
| Briefcase Carry | M Hook - BP | 7 (5.4, 8.6)*** | 7.6 (6, 9.2)*** | 15.1 (13.4, 16.8)*** | 6 (4.6, 7.4)*** | 7.6 (6.3, 9)*** | 15.1 (13.7, 16.6)*** | 6.1 (4.9, 7.3)*** | 7.6 (6.4, 8.9)*** | 15.4 (14.1, 16.8)*** |
|  | M Hand - BP | 11.8 (10.1, 13.5)*** | 13 (11.3, 14.7)*** | 20.9 (19.1, 22.8)*** | 10.6 (9.2, 12)*** | 13 (11.5, 14.4)*** | 21 (19.5, 22.6)*** | 10.9 (9.6, 12.1)*** | 13 (11.7, 14.2)*** | 21.5 (20.1, 22.9)*** |
|  | AP - BP | 30.6 (28.7, 32.6)*** | 32.3 (30.3, 34.3)*** | 49.1 (46.9, 51.3)*** | 25.2 (23.6, 26.8)*** | 32.3 (30.6, 34)*** | 49.3 (47.4, 51.3)*** | 24.4 (23, 25.8)*** | 32.3 (30.8, 33.8)*** | 50.5 (48.8, 52.2)*** |
|  | M Hand - M Hook | 4.5 (2.9, 6)*** | 4.9 (3.4, 6.5)*** | 5.1 (3.5, 6.7)*** | 4.4 (3, 5.7)*** | 4.9 (3.6, 6.3)*** | 5.1 (3.8, 6.5)*** | 4.5 (3.3, 5.7)*** | 4.9 (3.8, 6.1)*** | 5.2 (4.1, 6.4)*** |
|  | AP - M Hook | 22.1 (20.2, 23.9)*** | 22.9 (21.1, 24.8)*** | 29.6 (27.7, 31.5)*** | 18.1 (16.6, 19.7)*** | 22.9 (21.3, 24.5)*** | 29.7 (28.1, 31.4)*** | 17.3 (15.9, 18.6)*** | 22.9 (21.5, 24.3)*** | 30.4 (28.9, 31.8)*** |
|  | AP - M Hand | 16.8 (15.1, 18.6)*** | 17.1 (15.4, 18.9)*** | 23.3 (21.5, 25.1)*** | 13.2 (11.7, 14.7)*** | 17.1 (15.6, 18.6)*** | 23.4 (21.8, 25)*** | 12.2 (11, 13.5)*** | 17.1 (15.8, 18.4)*** | 23.9 (22.5, 25.3)*** |
| Jumping Jack | M Hook - BP | 195.7 (187.5, 204.1)*** | 330.1 (318.2, 342.4)*** | 91.5 (86.2, 97)*** | 216.6 (207.8, 225.7)*** | 330.1 (318.1, 342.4)*** | 94 (88.6, 99.5)*** | 272.3 (262.5, 282.4)*** | 330.4 (319, 342.1)*** | 111.6 (106, 117.3)*** |
|  | M Hand - BP | 314.7 (303.2, 326.5)*** | 566 (547.6, 585)*** | 131.7 (125.3, 138.3)*** | 353.6 (341, 366.6)*** | 566 (547.4, 585)*** | 135.1 (128.6, 141.9)*** | 453.9 (439.3, 469)*** | 566.4 (548.8, 584.6)*** | 159.5 (152.6, 166.5)*** |
|  | AP - BP | 717.7 (695.1, 741)*** | 1249.5 (1212.2, 1287.9)*** | 312.2 (300.8, 323.9)*** | 800.4 (775.3, 826.2)*** | 1249.5 (1212, 1288.2)*** | 320.3 (308.6, 332.4)*** | 1014 (984.5, 1044.2)*** | 1250.5 (1214.7, 1287.2)*** | 377.6 (365, 390.6)*** |
|  | M Hand - M Hook | 40.2 (36.4, 44.2)*** | 54.8 (50.6, 59.2)*** | 20.9 (17.6, 24.4)*** | 43.2 (39.3, 47.4)*** | 54.8 (50.5, 59.3)*** | 21.2 (17.9, 24.7)*** | 48.8 (44.8, 52.8)*** | 54.8 (50.7, 59)*** | 22.7 (19.4, 26)*** |
|  | AP - M Hook | 176.6 (168.9, 184.4)*** | 213.8 (205.1, 222.7)*** | 115.2 (109.2, 121.3)*** | 184.3 (176.4, 192.5)*** | 213.8 (205, 222.7)*** | 116.7 (110.7, 122.9)*** | 199.2 (191.3, 207.3)*** | 213.8 (205.5, 222.3)*** | 125.8 (119.8, 131.9)*** |
|  | AP - M Hand | 97.2 (91.7, 102.8)*** | 102.6 (97, 108.4)*** | 77.9 (73, 83)*** | 98.5 (93, 104.2)*** | 102.6 (97, 108.5)*** | 78.7 (73.8, 83.9)*** | 101.1 (95.8, 106.6)*** | 102.6 (97.3, 108.2)*** | 84.1 (79.2, 89.1)*** |
| Jug Lift | M Hook - BP | 11.7 (10.9, 12.5)*** | 7.8 (7, 8.6)*** | 15.6 (14.8, 16.4)*** | 10.8 (10, 11.6)*** | 7.8 (7.1, 8.6)*** | 15.7 (14.8, 16.5)*** | 9.7 (8.9, 10.5)*** | 7.8 (7.1, 8.6)*** | 16.1 (15.2, 16.9)*** |
|  | M Hand - BP | 17.7 (16.8, 18.5)*** | 13 (12.2, 13.8)*** | 22 (21.1, 22.8)*** | 16.7 (15.8, 17.5)*** | 13 (12.2, 13.8)*** | 22.1 (21.2, 23)*** | 15.4 (14.6, 16.3)*** | 13 (12.1, 13.8)*** | 22.6 (21.8, 23.5)*** |
|  | AP - BP | 42 (41, 43)*** | 29.4 (28.5, 30.4)*** | 52.6 (51.5, 53.7)*** | 39.4 (38.4, 40.4)*** | 29.5 (28.5, 30.4)*** | 52.8 (51.8, 54)*** | 36.1 (35.1, 37.1)*** | 29.5 (28.5, 30.4)*** | 54.2 (53.1, 55.3)*** |
|  | M Hand - M Hook | 5.4 (4.6, 6.2)*** | 4.8 (4, 5.5)*** | 5.5 (4.8, 6.3)*** | 5.3 (4.6, 6.1)*** | 4.8 (4, 5.5)*** | 5.5 (4.8, 6.3)*** | 5.3 (4.5, 6)*** | 4.8 (4, 5.5)*** | 5.7 (4.9, 6.4)*** |
|  | AP - M Hook | 27.2 (26.3, 28.1)*** | 20.1 (19.2, 20.9)*** | 32 (31.1, 32.9)*** | 25.8 (24.9, 26.7)*** | 20.1 (19.2, 20.9)*** | 32.1 (31.2, 33.1)*** | 24.1 (23.2, 25)*** | 20.1 (19.2, 20.9)*** | 32.9 (31.9, 33.8)*** |
|  | AP - M Hand | 20.7 (19.8, 21.5)*** | 14.6 (13.8, 15.4)*** | 25.1 (24.2, 26)*** | 19.5 (18.6, 20.3)*** | 14.6 (13.8, 15.4)*** | 25.2 (24.3, 26.1)*** | 17.9 (17, 18.7)*** | 14.6 (13.8, 15.4)*** | 25.7 (24.8, 26.7)*** |
| Jogging | M Hook - BP | 119.2 (105.8, 133.5)*** | 523 (484.8, 563.7)*** | 90.4 (78.8, 102.9)*** | 161.1 (145.4, 177.9)*** | 523 (485.4, 563)*** | 92.7 (81.1, 105.1)*** | 352.9 (326.2, 381.3)*** | 526.6 (489.6, 565.9)*** | 109 (96.7, 122.1)*** |
|  | M Hand - BP | 191.8 (173.9, 210.9)*** | 934.3 (870.9, 1001.9)*** | 127.6 (113.7, 142.5)*** | 285.5 (262.3, 310.3)*** | 934.3 (871.9, 1000.8)*** | 130.9 (117, 145.7)*** | 613.7 (571.6, 658.5)*** | 940.5 (879.1, 1005.8)*** | 153.9 (138.9, 169.8)*** |
|  | AP - BP | 472.9 (437.8, 510.4)*** | 2063.3 (1930.7, 2204.7)*** | 304.4 (279.6, 330.8)*** | 686.2 (638.7, 736.7)*** | 2063.3 (1932.8, 2202.3)*** | 312.1 (287.2, 338.6)*** | 1377.6 (1290.4, 1470.2)*** | 2075.7 (1947.3, 2212.1)*** | 366.7 (339.1, 396)*** |
|  | M Hand - M Hook | 33.1 (25, 41.8)*** | 66 (55.9, 76.9)*** | 19.5 (12.2, 27.4)*** | 47.6 (38.7, 57.1)*** | 66 (56, 76.7)*** | 19.8 (12.6, 27.5)*** | 57.6 (48.3, 67.5)*** | 66.1 (56.3, 76.5)*** | 21.5 (14.3, 29.1)*** |
|  | AP - M Hook | 161.4 (145.3, 178.4)*** | 247.3 (226, 270)*** | 112.3 (99.3, 126.2)*** | 201.1 (182.9, 220.4)*** | 247.3 (226.3, 269.6)*** | 113.8 (100.9, 127.6)*** | 226.2 (207, 246.7)*** | 247.2 (226.7, 269)*** | 123.3 (110.1, 137.3)*** |
|  | AP - M Hand | 96.3 (84.3, 109.1)*** | 109.2 (96.3, 122.8)*** | 77.6 (66.7, 89.2)*** | 103.9 (91.6, 117)*** | 109.2 (96.5, 122.6)*** | 78.5 (67.7, 90)*** | 107 (94.8, 120)*** | 109.1 (96.8, 122.2)*** | 83.8 (73, 95.4)*** |
| Underhand Toss | M Hook - BP | 196.4 (187.1, 206.1)*** | 298.1 (285.6, 311)*** | 111.1 (104.5, 118)*** | 225.1 (215.3, 235.3)*** | 297.4 (285.3, 309.8)*** | 111.6 (105.2, 118.2)*** | 279 (268.3, 290.1)*** | 300.2 (288.9, 311.8)*** | 127.9 (121.5, 134.6)*** |
|  | M Hand - BP | 318.8 (305.6, 332.4)*** | 517.8 (498.3, 537.8)*** | 164.5 (156.2, 173.1)*** | 370.2 (355.9, 384.8)*** | 516.6 (497.9, 535.9)*** | 165.3 (157.2, 173.5)*** | 467 (450.9, 483.5)*** | 521.3 (503.8, 539.4)*** | 189.4 (181.2, 197.8)*** |
|  | AP - BP | 728.1 (702.1, 755)*** | 1150.4 (1111, 1191)*** | 389.8 (374.4, 405.7)*** | 834.6 (806.3, 863.8)*** | 1147.9 (1110.2, 1186.9)*** | 391.3 (376.5, 406.7)*** | 1039.3 (1007.1, 1072.4)*** | 1156.9 (1121.3, 1193.4)*** | 446.9 (431.5, 462.8)*** |
|  | M Hand - M Hook | 41.3 (36.8, 45.9)*** | 55.2 (50.3, 60.2)*** | 25.3 (21.3, 29.4)*** | 44.6 (40.2, 49.1)*** | 55.2 (50.5, 60)*** | 25.3 (21.6, 29.3)*** | 49.6 (45.3, 53.9)*** | 55.3 (50.9, 59.8)*** | 27 (23.4, 30.7)*** |
|  | AP - M Hook | 179.4 (170.6, 188.4)*** | 214.1 (204.2, 224.3)*** | 132 (124.7, 139.5)*** | 187.5 (178.8, 196.4)*** | 214.1 (204.6, 223.9)*** | 132.2 (125.2, 139.4)*** | 200.6 (192.1, 209.3)*** | 214.1 (205.2, 223.2)*** | 139.9 (133.2, 146.9)*** |
|  | AP - M Hand | 97.7 (91.5, 104.2)*** | 102.4 (96, 109)*** | 85.2 (79.3, 91.2)*** | 98.8 (92.8, 105)*** | 102.4 (96.3, 108.7)*** | 85.2 (79.6, 91)*** | 100.9 (95.3, 106.8)*** | 102.3 (96.6, 108.2)*** | 89 (83.6, 94.5)*** |
| Internal Rotation | M Hook - BP | 216.9 (207.6, 226.6)*** | 328.3 (315.6, 341.4)*** | 104 (98, 110.2)*** | 215.4 (205.5, 225.6)*** | 328.6 (315.2, 342.5)*** | 107.5 (101, 114.3)*** | 238 (226.7, 249.7)*** | 328.6 (314.3, 343.4)*** | 128.6 (121, 136.6)*** |
|  | M Hand - BP | 358.2 (344.6, 372.2)*** | 578.3 (558.2, 599)*** | 157.1 (149.5, 164.9)*** | 356.6 (342.3, 371.4)*** | 578.8 (557.5, 600.7)*** | 162.4 (154.1, 170.9)*** | 405.9 (389, 423.4)*** | 578.8 (556, 602.3)*** | 193.9 (184.1, 204.1)*** |
|  | AP - BP | 820.5 (793.3, 848.7)*** | 1257.2 (1217, 1298.6)*** | 375 (360.9, 389.5)*** | 814.8 (786.2, 844.5)*** | 1258.2 (1215.6, 1302.1)*** | 387.1 (371.8, 402.8)*** | 918.4 (884.3, 953.6)*** | 1246.5 (1201.4, 1293.1)*** | 456.1 (437.5, 475.4)*** |
|  | M Hand - M Hook | 44.6 (40.3, 49)*** | 58.4 (53.7, 63.2)*** | 26 (22.3, 29.9)*** | 44.8 (40.3, 49.5)*** | 58.4 (53.4, 63.5)*** | 26.4 (22.5, 30.5)*** | 49.7 (44.7, 54.9)*** | 58.4 (53.1, 63.8)*** | 28.6 (24.3, 33)*** |
|  | AP - M Hook | 190.4 (181.8, 199.3)*** | 216.8 (207.5, 226.5)*** | 132.9 (126, 140)*** | 190.1 (181, 199.5)*** | 216.9 (206.9, 227.1)*** | 134.7 (127.3, 142.3)*** | 201.3 (191.2, 211.7)*** | 214.2 (203.6, 225)*** | 143.2 (135.1, 151.7)*** |
|  | AP - M Hand | 100.9 (95, 107)*** | 100.1 (94.2, 106.2)*** | 84.8 (79.3, 90.4)*** | 100.4 (94.1, 106.8)*** | 100.1 (93.8, 106.6)*** | 85.6 (79.8, 91.6)*** | 101.3 (94.6, 108.3)*** | 98.4 (91.7, 105.2)*** | 89.2 (82.9, 95.7)*** |

Notes: The percentage change in force measurement between each pairwise comparison of four models is reported;
95% Confidence Intervals of percentage changes are reported in parentheses;
$***$ indicates that p-value < 0.001; $**$ indicates that $0.001\leq p-value<0.01$; $*$ indicates that $0.01\leq p-value<0.05$;
“M Hook” stands for Myoelectric Hook, “BP” stands for Body Powered, “M Hand” stands for Myoelectric Hand, and “AP” stands for Advanced Prosthetic.

# Amputation level dependence on loading

Question: Is there a significant difference in kinetics between amputation lengths?

Hypothesis: Residual limb length will significantly impact the kinetics of the bone-implant interface.

**Table 3. Percentage Change in Force Measurement (Amputation Level 50% & 75% Compared to 25%)**

|  |  | Bending | | | Torsion | | | Axial | | |
| --- | --- | --- | --- | --- | --- | --- | --- | --- | --- | --- |
| Model | Motion | 50%vs.25% | 75%vs.25% | 75%vs.50% | 50%vs.25% | 75%vs.25% | 75%vs.50% | 50%vs.25% | 75%vs.25% | 75%vs.50% |
| Intact Arm | Briefcase Carry | -4.9 (-7.3, -2.5)*** | -1.8 (-4.2, 0.7) | 3.3 (0.8, 5.9)* | 0 (-2.5, 2.5) | 0 (-2.5, 2.5) | 0 (-2.5, 2.5) | -6 (-8.3, -3.6)*** | -12.1 (-14.2, -9.8)*** | -6.4 (-8.7, -4.1)*** |
| Body Powered |  | -2.9 (-4.9, -0.9)** | -1 (-3, 1.1) | 2 (-0.1, 4.2) | 0 (-2.1, 2.1) | 0 (-2.1, 2.1) | 0 (-2.1, 2.1) | -0.5 (-2.6, 1.6) | -3 (-5, -1)** | -2.5 (-4.5, -0.4)* |
| Myoelectric Hook |  | -3.9 (-6, -1.7)*** | -1.8 (-4, 0.4) | 2.1 (-0.2, 4.4) | 0 (-2.2, 2.3) | 0 (-2.2, 2.3) | 0 (-2.2, 2.3) | -0.5 (-2.7, 1.8) | -2.7 (-4.9, -0.5)* | -2.2 (-4.4, 0) |
| Myoelectric Hand |  | -4 (-6.1, -1.8)*** | -1.8 (-4, 0.4) | 2.2 (0, 4.6) | 0 (-2.2, 2.3) | 0 (-2.2, 2.3) | 0 (-2.2, 2.3) | -0.5 (-2.7, 1.8) | -2.6 (-4.7, -0.3)* | -2.1 (-4.3, 0.1) |
| Advanced Prosthetic |  | -7 (-9.4, -4.5)*** | -5.7 (-8.1, -3.2)*** | 1.4 (-1.3, 4.1) | 0 (-2.6, 2.7) | 0 (-2.6, 2.7) | 0 (-2.6, 2.7) | -0.4 (-2.9, 2.3) | -2.1 (-4.6, 0.5) | -1.8 (-4.3, 0.9) |
| Intact Arm | Jumping Jack | -20.3 (-21.4, -19.2)*** | -37.2 (-38.1, -36.3)*** | -21.2 (-22.3, -20.1)*** | -0.1 (-1.5, 1.3) | -0.2 (-1.6, 1.2) | -0.1 (-1.5, 1.3) | -14.4 (-15.6, -13.2)*** | -29.3 (-30.3, -28.3)*** | -17.4 (-18.5, -16.2)*** |
| Body Powered |  | -25.1 (-26.4, -23.8)*** | -51.2 (-52.1, -50.4)*** | -34.9 (-36, -33.8)*** | 0 (-1.7, 1.7) | -0.1 (-1.8, 1.7) | -0.1 (-1.8, 1.7) | -2.6 (-4.2, -0.9)** | -17.2 (-18.6, -15.8)*** | -15 (-16.5, -13.6)*** |
| Myoelectric Hook |  | -19.8 (-20.8, -18.7)*** | -38.6 (-39.4, -37.8)*** | -23.4 (-24.4, -22.4)*** | 0 (-1.3, 1.3) | 0 (-1.3, 1.3) | 0 (-1.3, 1.3) | -1.3 (-2.6, 0)* | -8.6 (-9.8, -7.4)*** | -7.3 (-8.5, -6.1)*** |
| Myoelectric Hand |  | -18.1 (-19.1, -17.1)*** | -34.9 (-35.6, -34.1)*** | -20.5 (-21.4, -19.5)*** | 0 (-1.2, 1.2) | 0 (-1.2, 1.2) | 0 (-1.2, 1.2) | -1.1 (-2.3, 0.1) | -7.3 (-8.4, -6.1)*** | -6.2 (-7.4, -5.1)*** |
| Advanced Prosthetic |  | -17.5 (-18.5, -16.6)*** | -33.6 (-34.3, -32.8)*** | -19.5 (-20.4, -18.5)*** | 0 (-1.1, 1.1) | 0 (-1.1, 1.1) | 0 (-1.1, 1.1) | -0.6 (-1.8, 0.5) | -4.1 (-5.1, -3)*** | -3.4 (-4.5, -2.3)*** |
| Intact Arm | Jug Lift | -14.5 (-15, -14.1)*** | -27.6 (-28, -27.2)*** | -15.3 (-15.7, -14.9)*** | 0 (-0.5, 0.5) | 0 (-0.5, 0.5) | 0 (-0.5, 0.5) | -6.1 (-6.6, -5.7)*** | -12.3 (-12.8, -11.9)*** | -6.6 (-7, -6.1)*** |
| Body Powered |  | -12.2 (-12.7, -11.8)*** | -24.3 (-24.6, -23.9)*** | -13.7 (-14.1, -13.3)*** | 0 (-0.5, 0.5) | 0 (-0.5, 0.5) | 0 (-0.5, 0.5) | -0.6 (-1, -0.1)* | -3.2 (-3.6, -2.7)*** | -2.6 (-3.1, -2.2)*** |
| Myoelectric Hook |  | -12.9 (-13.3, -12.5)*** | -25.6 (-26, -25.3)*** | -14.6 (-15, -14.2)*** | 0 (-0.5, 0.5) | 0 (-0.5, 0.5) | 0 (-0.5, 0.5) | -0.5 (-1, 0)* | -2.8 (-3.2, -2.3)*** | -2.3 (-2.8, -1.8)*** |
| Myoelectric Hand |  | -13 (-13.4, -12.6)*** | -25.7 (-26.1, -25.3)*** | -14.6 (-15, -14.2)*** | 0 (-0.5, 0.5) | 0 (-0.5, 0.4) | 0 (-0.5, 0.4) | -0.5 (-0.9, 0) | -2.6 (-3.1, -2.2)*** | -2.2 (-2.6, -1.7)*** |
| Advanced Prosthetic |  | -13.9 (-14.3, -13.4)*** | -27.4 (-27.8, -27.1)*** | -15.7 (-16.1, -15.3)*** | 0 (-0.5, 0.5) | 0 (-0.5, 0.5) | 0 (-0.5, 0.5) | -0.4 (-0.8, 0.1) | -2.1 (-2.6, -1.6)*** | -1.7 (-2.2, -1.3)*** |
| Intact Arm | Jogging | -12.5 (-15.4, -9.5)*** | -2.1 (-5.3, 1.3) | 12 (8.3, 15.8)*** | 0 (-3.3, 3.4) | 0 (-3.3, 3.4) | 0 (-3.3, 3.4) | -14.5 (-17.3, -11.6)*** | -29.7 (-32, -27.3)*** | -17.8 (-20.5, -14.9)*** |
| Body Powered |  | -32.8 (-34.5, -31)*** | -61.2 (-62.2, -60.1)*** | -42.3 (-43.8, -40.7)*** | 0 (-2.6, 2.7) | -0.6 (-3.2, 2.1) | -0.6 (-3.2, 2.1) | -2.5 (-5, 0.2) | -16.9 (-19.1, -14.7)*** | -14.8 (-17.1, -12.5)*** |
| Myoelectric Hook |  | -19.9 (-22.3, -17.5)*** | -19.8 (-22.2, -17.4)*** | 0.1 (-2.9, 3.2) | 0 (-3, 3.1) | 0 (-3, 3.1) | 0 (-3, 3.1) | -1.3 (-4.2, 1.7) | -8.8 (-11.5, -6)*** | -7.6 (-10.4, -4.8)*** |
| Myoelectric Hand |  | -11.2 (-13.9, -8.4)*** | -5.1 (-7.9, -2.1)*** | 6.9 (3.7, 10.2)*** | 0 (-3, 3.1) | 0 (-3, 3.1) | 0 (-3, 3.1) | -1.1 (-4.1, 2) | -7.4 (-10.2, -4.5)*** | -6.4 (-9.2, -3.4)*** |
| Advanced Prosthetic |  | -7.8 (-10.4, -5)*** | 0.1 (-2.8, 3) | 8.5 (5.4, 11.7)*** | 0 (-2.9, 3) | 0 (-2.9, 2.9) | 0 (-2.9, 2.9) | -0.6 (-3.5, 2.3) | -4.1 (-6.9, -1.3)** | -3.5 (-6.3, -0.7)* |
| Intact Arm | Underhand Toss | -15.7 (-17.3, -14.1)*** | -28.8 (-30.1, -27.4)*** | -15.5 (-17.1, -13.9)*** | -0.2 (-2.1, 1.7) | -0.4 (-2.3, 1.5) | -0.2 (-2.1, 1.7) | -9.1 (-10.8, -7.4)*** | -20 (-21.4, -18.4)*** | -11.9 (-13.5, -10.2)*** |
| Body Powered |  | -23.9 (-25.4, -22.5)*** | -46.8 (-47.8, -45.8)*** | -30 (-31.4, -28.7)*** | 0.2 (-1.7, 2.1) | -0.5 (-2.4, 1.4) | -0.7 (-2.6, 1.2) | -0.3 (-2.2, 1.6) | -12.4 (-14.1, -10.8)*** | -12.2 (-13.8, -10.5)*** |
| Myoelectric Hook |  | -16.6 (-18.1, -15)*** | -32 (-33.2, -30.6)*** | -18.4 (-20, -16.9)*** | 0 (-1.9, 1.9) | 0 (-1.9, 1.9) | 0 (-1.9, 1.9) | -0.1 (-2, 1.8) | -5.5 (-7.2, -3.7)*** | -5.4 (-7.2, -3.6)*** |
| Myoelectric Hand |  | -14.6 (-16.1, -13)*** | -27.9 (-29.2, -26.6)*** | -15.6 (-17.1, -14.1)*** | 0 (-1.8, 1.8) | 0 (-1.7, 1.8) | 0 (-1.7, 1.8) | 0 (-1.8, 1.8) | -4.2 (-5.9, -2.5)*** | -4.2 (-5.9, -2.4)*** |
| Advanced Prosthetic |  | -14.1 (-15.6, -12.6)*** | -26.8 (-28.1, -25.5)*** | -14.7 (-16.2, -13.2)*** | 0 (-1.8, 1.8) | 0 (-1.8, 1.8) | 0 (-1.7, 1.8) | 0 (-1.8, 1.8) | -2.2 (-3.9, -0.5)* | -2.2 (-3.9, -0.5)* |
| Intact Arm | Internal Rotation | -33 (-36, -29.9)*** | -61.5 (-63.2, -59.7)*** | -42.6 (-45.1, -39.9)*** | 5 (0.3, 9.9)* | -0.2 (-4.6, 4.4) | -4.9 (-9.1, -0.5)* | -12 (-15.9, -7.9)*** | -23.7 (-27.1, -20.1)*** | -13.3 (-17.2, -9.3)*** |
| Body Powered |  | -31.4 (-32.7, -30)*** | -63.3 (-64, -62.6)*** | -46.5 (-47.5, -45.5)*** | -0.1 (-2, 1.9) | -0.1 (-2, 1.9) | 0 (-1.9, 1.9) | -3 (-4.8, -1.1)** | -17.6 (-19.2, -16)*** | -15.1 (-16.7, -13.4)*** |
| Myoelectric Hook |  | -31.7 (-33.6, -29.8)*** | -60.9 (-62, -59.8)*** | -42.7 (-44.3, -41.1)*** | 0 (-2.8, 2.8) | 0 (-2.8, 2.8) | 0 (-2.8, 2.8) | -1.3 (-4, 1.5) | -7.6 (-10.2, -5)*** | -6.4 (-9, -3.8)*** |
| Myoelectric Hand |  | -31.6 (-33.6, -29.6)*** | -59.5 (-60.7, -58.3)*** | -40.8 (-42.5, -39)*** | 0 (-2.9, 3) | 0 (-2.9, 3) | 0 (-2.9, 3) | -1 (-3.8, 2) | -5.8 (-8.5, -3)*** | -4.8 (-7.6, -2)** |
| Advanced Prosthetic |  | -31.8 (-33.9, -29.6)*** | -59.4 (-60.7, -58.1)*** | -40.5 (-42.3, -38.6)*** | 0 (-3.1, 3.2) | -0.9 (-3.9, 2.3) | -0.9 (-3.9, 2.3) | -0.5 (-3.6, 2.7) | -3.5 (-6.5, -0.4)* | -3 (-6, 0.1) |

Notes: The percentage change in force measurement between current amputation level and 25% amputation level is reported;
95% Confidence Intervals of percentage changes are reported in parentheses;
$***$ indicates that p-value < 0.001; $**$ indicates that $0.001\leq p-value<0.01$; $*$ indicates that $0.01\leq p-value<0.05$.
